# Elementary Anatomy, in Sixteen Plates

**Published:** 1849-05

**Authors:** 


*Elementary Anatomy, in Sixteen Plates:*—Representing the full-length human figure, half the size of life. Together with a separate explanatory text. The whole forming a concise Manual of Physiological Anatomy. Intended for the use of Physicians, Medical Students, Naturalists, Painters, Sculptors, Lectures on Anatomy, Schools, Families, and in general for all who wish readily to acquire a knowledge of the organization of the human body. From the French of Bourgery and Jacob. The text translated by J. C. Comstock, A. M. Kelloggs and Comstock, Publishers, 150 Fulton Street, New York, and 136 Main Street, Hartford—1849.

We have examined the Anatomical plates of which the above is the title, and, in our opinion, they are scarcely, if at all, inferior, to those in the celebrated work of M. M. Bourgery and Jacob, entitled, "*Anatomie Elementaire,*" of which they are copies. They are furnished at one half the price of the French plates. We would commend them to Medical Students, Practitioners, and others, as affording valuable aid in the study and review of Anatomy.

